# Superimposed CSI Feedback Assisted by Inactive Sensing Information

**DOI:** 10.3390/s25196156

**Published:** 2025-10-04

**Authors:** Mintao Zhang, Haowen Jiang, Zilong Wang, Linsi He, Yuqiao Yang, Mian Ye, Chaojin Qing

**Affiliations:** 1 School of Electrical Engineering and Electronic Information, Xihua University, Chengdu 610039, China; zhangmt@mail.xhu.edu.cn (M.Z.); jhw641300431@163.com (H.J.); wan_zilong@163.com (Z.W.); helinsr@163.com (L.H.); 18716138509@163.com (Y.Y.); 2 School of Aeronautics and Astronautics, Xihua University, Chengdu 610039, China

**Keywords:** channel state information (CSI), superimposed CSI feedback, inactive sensing information, sensing-assisted communication, delay–Doppler (DD) domain

## Abstract

In massive multiple-input and multiple-output (mMIMO) systems, superimposed channel state information (CSI) feedback is developed to improve the occupation of uplink bandwidth resources. Nevertheless, the interference from this superimposed mode degrades the recovery performance of both downlink CSI and uplink data sequences. Although machine learning (ML)-based methods effectively mitigate superimposed interference by leveraging the multi-domain features of downlink CSI, the complex interactions among network model parameters cause a significant burden on system resources. To address these issues, inspired by sensing-assisted communication, we propose a novel superimposed CSI feedback method assisted by inactive sensing information that previously existed but was not utilized at the base station (BS). To the best of our knowledge, this is the first time that inactive sensing information is utilized to enhance superimposed CSI feedback. In this method, a new type of modal data, different from communication data, is developed to aid in interference suppression without requiring additional hardware at the BS. Specifically, the proposed method utilizes location, speed, and path information extracted from sensing devices to derive prior information. Then, based on the derived prior information, denoising processing is applied to both the delay and Doppler dimensions of downlink CSI in the delay—Doppler (DD) domain, significantly enhancing the recovery accuracy. Simulation results demonstrate the performance improvement of downlink CSI and uplink data sequences when compared to both classic and novel superimposed CSI feedback methods. Moreover, against parameter variations, simulation results also validate the robustness of the proposed method.

## 1. Introduction

Massive multiple-input and multiple-output (mMIMO) is widely recognized as a key technique in fifth-generation (5G) communications for its high spectrum and energy efficiency [1,2,3]. The benefits of MIMO systems heavily rely on the accuracy of downlink channel state information (CSI) obtained by the base station (BS) [4]. In frequency division duplex (FDD) systems, the downlink CSI estimated by user equipment (UE) usually needs to be fed back to the BS [5,6]. This inevitably causes huge feedback overhead, attributed to the substantial number of BS antennas that heavily occupy bandwidth resources [7].

To avoid the extra bandwidth occupation, a superimposed CSI feedback method is developed in [8]. The downlink CSI is superimposed on the uplink data sequences (UL-DS) to feed back to the BS, avoiding occupying uplink bandwidth resources and thus incurring no feedback overhead. Consequently, the cost of CSI feedback is minimal, leading to a substantial enhancement of the system’s spectral efficiency [9]. Nonetheless, the superimposed mode inevitably introduces the superimposed interference, deteriorating the recovery performance of downlink CSI and UL-DS [10]. Therefore, effectively suppressing superimposed interference has emerged as an urgent issue for resolving challenges associated with superimposed CSI feedback [11].

For superimposed interference suppression (SIS) and recovery performance improvement, machine learning (ML)-based methods of superimposed CSI feedback are developed in [12,13,14]. These ML-based methods exploit the coherence of multiple domains from the downlink CSI [15]. In [12], an extreme learning machine-based method is investigated for superimposed CSI feedback in the uplink communication process. In [13], a neural network framework is proposed in which the UE extracts and superimposes CSI features onto images, and the BS recovers the CSI from transmitted images through deep learning. In [14], convolutional neural networks are adopted to extract CSI features, superimpose CSI in audio signals, and reconstruct CSI from the audio signals. Although these methods have partially mitigated superimposed interference, they typically involve a substantial interaction of network model parameters between the BS and UE. For example, during initial deployment, BS must transmit the model architecture/weights to UE. During online operation, UE may upload locally fine-tuned gradients, or BS broadcasts updated weights to UEs when channel distribution shifts [3]. This interaction consumes substantial bandwidth, contradicting the goal of reducing feedback overhead and complicating practical implementation [16]. A critical question emerges as to whether there is a straightforward method that can be developed and utilized for superimposed CSI feedback without imposing a significant burden on system resources.

In actuality, whether applied or not, many BSs are equipped with a significant number of sensing devices, such as radar and global positioning systems (GPSs) [17,18]. Prior to the establishment of communication links, these sensing devices can gather a wealth of useful information that could significantly enhance communication performance. However, this potential for performance improvement remains largely unrealized, resulting in a vast amount of inactive sensing information. It refers to sensory data (e.g., location, speed, and path information) already available at the BS through embedded devices prior to communication link establishment. Such information is passively acquired without an extra hardware overhead but remains underutilized for communication tasks. Therefore, to suppress superimposed interference during CSI recovery and fully leverage the capabilities of these sensors, inactive sensing information should be developed as additional modal data. Notably, the recently proposed sensing-assisted communication is expected to provide improvement in the performance of communication-centric applications [19]. Many applications of sensing-assisted communication have been proposed, such as sensing-assisted beam training [20], sensing-assisted beam tracking and prediction [21], sensing-assisted channel estimation [22], etc. In contrast, few studies investigate sensing-assisted CSI feedback methods, nor has there been significant effort dedicated to leveraging this inactive sensing information, despite the potential for achieving high-precision sensing information without incurring extra hardware overheads [23].

Inspired by the idea of sensing-assisted communication [20,21,22], a superimposed CSI feedback method with inactive sensing information assistance is proposed in this paper. In this work, sensing information is developed to suppress transceiver noise and superimposed interference, thereby assisting superimposed CSI feedback and improving CSI reconstruction accuracy. Unlike classic sensing-assisted communication applications, the proposed method exploits the inactive sensing information that was previously existing but not utilized at the BS. This enables the generation of prior knowledge, thereby improving the recovery performance of downlink CSI reconstruction. Specifically, we utilize the inactive sensing information to acquire location, speed, and path information at the BS. Subsequently, we derive prior information in both delay and Doppler dimensions for superimposed CSI feedback. Leveraging the sensing prior information, we conduct denoising processing in both the delay and Doppler dimensions, thereby enhancing the recovery accuracy. It should be noted that the proposed method is also applicable to ML-based superimposed CSI feedback. To focus on the exploitation and augmentation of inactive sensing information, we validate the effectiveness of the ML-based methods by using the simulation approach. In particular, this paper merely serves as an example of utilizing inactive sensing information. We employ the sensing information of location, speed, and path to demonstrate the methodology of sensing-based CSI feedback. The incorporation of additional sensing information has the potential to further enhance the accuracy of CSI recovery.

The main contributions of this paper are summarized as follows:

1. We introduce the inactive sensing information into the superimposed CSI feedback, opening up a new mode of sensing information-assisted superimposed CSI feedback. Thus, the forgotten sensing information is utilized in the superimposed CSI feedback. To the best of our knowledge, this work presents the first leverage of inactive sensing information to assist superimposed CSI feedback. Moreover, this mode can be easily extended to other inactive sensing information-assisted communication.
2. We derive prior information for interference suppression in superimposed CSI feedback by leveraging the inactive sensing information. In the paper, we take the UE and BS embedded global navigation satellite system (GNSS) chips as an example to derive prior information in both delay and Doppler dimensions. As a result, a new modal data different from communication signals can be obtained for CSI feedback, forming additional prior information for SIS.
3. We propose a sensing information-assisted superimposed CSI feedback method. Based on the introduction of sensing information and the derivation of prior information, the CSI recovery in superimposed CSI feedback can be further denoised in the delay–Doppler (DD) domain. In this method, a denoising process is developed by leveraging sensing prior information. This only requires simple processing and slightly increased computational complexity, while achieving a significant performance improvement in the SIS.

The rest of this paper is structured as follows. In Section 2, we present the sensing information-assisted CSI feedback system. The sensing information-assisted superimposed CSI feedback method is presented in Section 3 and followed by the computational complexity analysis in Section 4. Subsequently, the experiment results are provided in Section 5. Finally, Section 6 concludes our work.

*Notations*: Boldface upper case and lower case letters denote matrix and vector, respectively; ${\left(\cdot \right)}^{T}$ and ${\left(\cdot \right)}^{H}$ denote transpose and Hermitian transpose, respectively; $I_{P}$ is the identity matrix of size P×P; $H\left(k,:\right)$ refers to the *k*-th row vector of H, $H\left(:,a:b\right)$ is the sub-matrix containing the *a*-th to *b*-th columns of H, and $H\left(:,:,c\right)$ is the *c*-th sub-matrix of H; $\left\lceil \cdot \right\rceil$ represents the operation of round up; $\mathrm{vec}\left(\cdot \right)$ denotes the vectorizing of a matrix, and $\mathrm{ve}c^{-1}\left(\cdot \right)$ is the corresponding inverse operation; ⊙ and ⊗ denote the Hadamard product and Kronecker product, respectively; $0_{a\times b}$ and $1_{a\times b}$ represent the all zeros and ones matrix of size a×b, respectively.

## 2. CSI Feedback System

**Figure 1:**
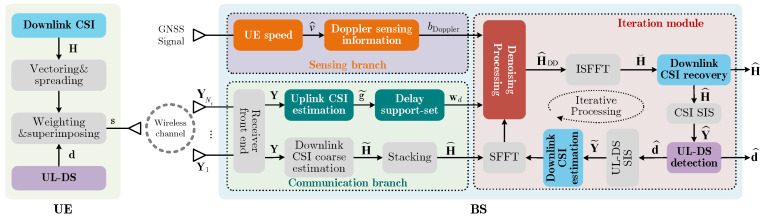
Sensing information-assisted CSI feedback system.

The sensing information-assisted CSI feedback system is depicted in Figure 1. In this system, the BS employs a uniform linear array (ULA) with $N_{t}$ antennas to serve a single-antenna UE moving at speed *v*. This paper focuses primarily on sensing-assisted CSI feedback. Thus, the channel estimation method and the design of the pilot pattern in [24,25] are employed (without a dedicated design for them). Operating in orthogonal frequency division multiplexing (OFDM) systems with *M* subcarriers, the estimated downlink channel vector between the BS and UE of the *m*-th subcarrier is denoted as $h_{m}\in C^{N_{t}\times 1}$, with m=1, 2,…,M. The downlink CSI matrix in the spatial–frequency domain is expressed as $H=\left[h_{1},h_{2},\ldots ,h_{M}\right]\in C^{N_{t}\times M}$, which is then vectorized to form the downlink CSI vector $x\in C^{1\times N_{t}M}$ according to $x=\mathrm{vec}\left(H\right)$. The vector x is subsequently spread and superimposed on the UL-DS to generate the transmitted signal $s\in C^{1\times N_{d}}$, which is provided by [8]:

(1) s=1−ρEd+ρEρENtMNtMxQT,

where $\rho \in \left[0,1\right]$ stands for the power proportional coefficient of the downlink CSI (i.e., a larger ρ means more power is allocated to the downlink CSI, resulting in less interference from UL-DS and higher reconstruction accuracy of the downlink CSI), *E* represents the transmitted power of UE, $d\in C^{1\times N_{d}}$ is the modulated UL-DS with the length of $N_{d}$, and $Q\in R^{N_{d}\times N_{t}M}$ is the spread spectrum matrix satisfying that $Q^{T}Q=N_{d}I_{N_{t}M}$.

At the BS, the received uplink signal at the *m*-th subcarrier, denoted as $Y_{m}\in C^{N_{t}\times N_{d}}$, is provided by

(2) $$\begin{array}{cccc} & & Y_{m}=g_{m}s_{m}+V_{m}, & \end{array}$$

where $g_{m}\in C^{N_{t}\times 1}$, $s_{m}\in C^{1\times N_{d}}$, and $V_{m}\in C^{N_{t}\times N_{d}}$ denote the uplink channel between the UE and BS, the transmitted signal, and the noise matrix of the *m*-th subcarrier, respectively. For the convenience of expression and to facilitate the subsequent derivations, we omit the subscript *m* and rewrite Equation (2) as

(3) $$\begin{array}{cccc} & & Y=gs+V. & \end{array}$$

In Equation (3), each entry of V is circularly symmetric complex Gaussian (CSCG) noise with zero-mean and variance $\sigma^{2}$. Based on Y, the inactive sensing information (e.g., GNSS information) is extracted and employed to assist in the recovery of downlink CSI H and UL-DS d, as described in Section 3.

## 3. Sensing Information-Assisted CSI Feedback

In this section, we present the proposed superimposed CSI feedback by leveraging the inactive sensing information. In Section 3.1 and Section 3.2, the Doppler shift and path delay prior information are sensed from GNSS data and uplink transmission, respectively. With the prior information, the recovery of downlink CSI and UL-DS is elaborated in Section 3.3.

### 3.1. Doppler Shift Prior Using Speed Sensing

UE locations have been available in the cellular network since the development second-generation (2G) wireless communication systems [26]; they are particularly relevant when emergency calls are made. Additionally, the majority of existing BSs and UEs are equipped with GNSS chips [27,28,29]. In practice, GNSS data are actually available at the UE through its GNSS chipset. Furthermore, such data are usually reported to the BS via existing control signaling (e.g., radio resource control (RRC) measurement) [29]. In particular, this data is time-aligned using the UE ID and a time stamp, requiring no extra hardware or air-interface overhead. Consequently, BSs can readily access various sensing information of UEs. The GNSS data provide accurate location and speed sensing information, which is then utilized to estimate the Doppler shift prior information. In this paper, we take the location and speed sensing information as an example to illustrate the substantial advantages it offers for CSI feedback.

At the moments *t* and t+Δt, the location coordinates of the UE can be denoted as $\left(x_{t},\;y_{t}\right)$ and $\left(x_{t+\Delta t},\;y_{t+\Delta t},\right)$, respectively [30]. The magnitude of displacement $R_{\Delta t}$ within the time interval Δt is calculated by

(4) $$\begin{array}{cccc} & & R_{\Delta t}=\sqrt{{\left(x_{t+\Delta t}-x_{t}\right)}^{2}+{\left(y_{t+\Delta t}-y_{t}\right)}^{2}}. & \end{array}$$

For a short time interval, Δt, it can be considered that the UE is moving in a straight direction and the magnitude of displacement is approximately equal to its traveled distance. Consequently, the UE speed is estimated by

(5) $$\begin{array}{cccc} & & \hat{v}=\frac{R_{\Delta t}}{\Delta t}, & \end{array}$$

where $\hat{v}$ denotes the sensed speed. According to [31], the maximum Doppler shift is determined by the carrier frequency and the UE speed. By denoting the maximum Doppler shift as $\nu_{\max}$, it is provided by [31]

(6) $$\begin{array}{cccc} & & \nu_{\max}=\frac{\hat{v}f_{c}}{c}, & \end{array}$$

where $f_{c}$ and *c* are the downlink carrier frequency and speed of light, respectively. To perform denoising processing, the continuous Doppler frequency shift is sampled and quantized according to the discrete grid in the DD domain with the Doppler shift resolution 11NTNT [32,33]. In the DD domain, the maximum Doppler frequency shift $\nu_{\max}$ is in correspondence with a specific boundary value $b_{\mathrm{Doppler}}$ positioned along the Doppler axis, and this association is mathematically expressed as

(7) bDoppler=νmax11NTNT,

where 11NTNT is the resolution of the path Doppler shifts, with *N* and *T* being the number of OFDM symbols and symbol duration, respectively [32,33]. It is worth noting that the proposed method utilizes the Doppler shift grid boundary value $b_{\mathrm{Doppler}}$ in the DD domain, instead of requiring exact path Doppler shift indices. Even if the UE location coordinates provided by GNSS contain errors, as long as the time interval Δt is accurate, the grid index of the maximum Doppler frequency shift still falls into the same DD grid cell, and the true Doppler frequency indices remain within the bounds of $b_{\mathrm{Doppler}}$.

With the Doppler shift prior information derived by the sensed speed, we further leverage the path-delay reciprocity between uplink CSI and downlink CSI to help downlink CSI recovery, which is elaborated in Section 3.2.

### 3.2. Path Delay Prior from Uplink CSI

In the uplink transmission, the BS can sense and acquire the uplink CSI. Based on this, we can derive the path delay prior information of the downlink CSI from the sensed uplink CSI.

At the BS, the uplink CSI of the *m*-th subcarrier (i.e., $g_{m}$ in Equation (2)) is obtained according to uplink channel estimation. The uplink CSI matrix in the spatial–frequency domain, denoted as $G\in C^{N_{t}\times M}$, is expressed by

(8) $$\begin{array}{cccc} & & G=\left[g_{1},g_{2},\ldots ,g_{M}\right]. & \end{array}$$

Subsequently, we employ the inverse discrete Fourier transform (IDFT) to transform the estimated G from the frequency domain to the delay domain [34]. By denoting this delay domain variant as $\hat{G}\in C^{N_{t}\times M}$, we have

(9) $$\begin{array}{cccc} & & \hat{G}={\mathrm{GF}}_{M}^{H}, & \end{array}$$

where $F_{M}$ is the M×M discrete Fourier transform (DFT) matrix. According to [35], by borrowing the expression of compressed sensing, a support set $W_{\mathrm{ul}}\in {\left\{0,1\right\}}^{N_{t}\times M}$ is employed to index the zero and non-zero entries of $\hat{G}$, i.e.,

(10) $$\begin{array}{cccc} & & W_{\mathrm{ul}}=\mathrm{supp}\left(\hat{G}\right), & \end{array}$$

where $\mathrm{supp}\left(\cdot \right)$ represents the operation of finding the support set of $\hat{G}$. According to [36], the zero elements and non-zero elements in $\hat{G}$ are labeled as 0 and 1, respectively. Since the receiving antennas at the BS are centralized, the delay support sets of the uplink CSI transmitted from the UE to each BS receiving antenna are almost identical due to the similar transmission paths. By extracting a row from the support set $W_{\mathrm{ul}}$, the delay support set of the uplink CSI for the $n_{t}$-th BS receiving antenna with $n_{t}=1,2,\cdots ,N_{t}$, denoted as $w_{\mathrm{ul},n_{t}}\in {\left\{0,1\right\}}^{1\times M}$, is obtained by

(11) $$\begin{array}{cccc} & & w_{\mathrm{ul},n_{t}}=W_{\mathrm{ul}}\left(n_{t},:\right). & \end{array}$$

From [35], there exists a path-delay reciprocity between the uplink and downlink CSIs due to the shared common physical paths and similar spatial propagation characteristics. That is, the uplink and downlink CSIs can share a similar delay support set during a downlink and uplink communication round. By denoting the support set of downlink CSI from the $n_{t}$-th transmitting BS antenna in the delay domain as $w_{\mathrm{dl},n_{t}}\in {\left\{0,1\right\}}^{1\times M}$, we have

(12) $$\begin{array}{cccc} & & w_{\mathrm{dl},n_{t}}=w_{\mathrm{ul},n_{t}}+\Delta w_{n_{t}}, & \end{array}$$

where $\Delta w_{n_{t}}$ denotes the difference between the delay support set of downlink and uplink CSI. Similar to the consideration in [37], the delay support sets of uplink and downlink CSI can be viewed as the same in a downlink and uplink communication duration, i.e., $\Delta w_{n_{t}}=0_{1\times M}$.

In this paper, we view $b_{\mathrm{Doppler}}$ in Equation (7) and $\left\{w_{\mathrm{dl},n_{t}}\right\}$ provided in Equation (12) as the prior information of the Doppler shift and path delay, respectively. These pieces of derived prior information serve as crucial factors, significantly assisting in downlink CSI recovery and UL-DS detection. The detailed algorithm for sensing and deriving prior information is summarized in Algorithm 1.
**Algorithm 1** Doppler shift and path delay prior derivations.***Input*****:**   Location coordinates $\left(x_{t},\;y_{t}\right)$, $\left(x_{t+\Delta t},\;y_{t+\Delta t},\right)$, time interval Δt, and uplink CSI $g_{m}$.**Derive the Doppler shift prior information:**1: Utilize GNSS signals to obtain UE location coordinates $\left(x_{t},\;y_{t}\right)$ and $\left(x_{t+\Delta t},\;y_{t+\Delta t},\right)$ at the moments *t* and t+Δt, respectively.2: Calculate the magnitude of displacement/distance $R_{\Delta t}$ within the time interval Δt according to Equation (4).3: Estimate the UE speed by using Equation (5).4: Obtain the maximum Doppler shift $\nu_{\max}$ via Equation (6).5: Derive a specific boundary value $b_{\mathrm{Doppler}}$ as the Doppler shift prior information by utilizing Equation (7).**Derive the path delay prior information:**1: Utilize communication signals to estimate the uplink CSI $g_{m}$ of the *m*-th subcarrier.2: Obtain the spatial–frequency uplink CSI matrix G via Equation (8).3: Transform the uplink CSI G to the delay domain by using Equation (9).4: Calculate the uplink CSI support set $W_{\mathrm{ul}}$ according to Equation (10).5: Extract the delay support set $w_{\mathrm{ul},n_{t}}$ through Equation (11).6: Derive a downlink CSI delay support set $\left\{w_{\mathrm{dl},n_{t}}\right\}$ as the path delay prior information by utilizing Equation (12).***Output*****:**  Doppler shift prior information, $b_{\mathrm{Doppler}}$, and path delay prior information, $\left\{w_{\mathrm{dl},n_{t}}\right\}$.

**Remark** **1.** *It is worth noting that $b_{\mathrm{Doppler}}$ is utilized as the Doppler shift prior information to assist the downlink CSI recovery. Other inactive sensing information can also be employed to derive prior information for CSI feedback. Accordingly, we use the delay support set as an example to illustrate prior information for the proposed method. In this paper, the delay support sets of each antenna are considered equal for the simplification consideration. Although other support sets (e.g., the angular support set) could be exploited, they are not further elaborated due to their evident resemblance to the provided example. In this paper, we view $b_{\mathrm{Doppler}}$ and $\left\{w_{\mathrm{dl},n_{t}}\right\}$ as the Doppler shift and path delay prior information to assist downlink CSI recovery and UL-DS detection.*

Subsequently, the prior information-assisted downlink CSI recovery and UL-DS detection are elaborated in Section 3.3, wherein no machine learning-based method is employed. Nonetheless, it is worth noting that emerging ML-based CSI feedback methods (e.g., [12]) can also incorporate the methodology proposed in this paper. We validate this adaptation by using a simulation.

### 3.3. Downlink CSI Recovery and UL-DS Detection

To recover the downlink CSI, we employ classic MMSE estimation to obtain a preliminary downlink CSI denoted as $\tilde{x}\in C^{1\times N_{t}M}$, as provided in [8]:

(13) $$\begin{array}{cccc} & & \tilde{x}=\sqrt{\frac{N_{t}M}{\rho E}}\frac{1}{N_{d}}{\left(\beta g^{H}g+\frac{N_{t}M}{\rho EN_{d}}\sigma^{2}\right)}^{-1}g^{H}\mathrm{YQ}, & \end{array}$$

where $\beta =1+\frac{\left(1-\rho \right)N_{t}M}{\rho N_{d}}$, and it is employed to simplify the expression. Subsequently, $\tilde{x}$ is transferred to the downlink CSI matrix $\tilde{H}\in C^{N_{t}\times M}$ in the time domain according to $\tilde{H}=\mathrm{ve}c^{-1}\left(\tilde{x}\right)$. By continuously stacking *N* downlink CSI matrices, a three-dimensional matrix $\overset{⌢}{H}\in C^{N_{t}\times M\times N}$ is formulated, which is expressed as     

(14) $$\begin{array}{cccc} & & \overset{⌢}{H}=\left[{\overset{⌢}{H}}_{1},{\overset{⌢}{H}}_{2},\ldots ,{\overset{⌢}{H}}_{Nt}\right], & \end{array}$$

where ${\overset{⌢}{H}}_{nt}\in C^{M\times N}$ represents the time-frequency (TF) domain channel corresponding to the $n_{t}$-th antenna at the BS with $n_{t}=1,2,\ldots ,N_{t}$. Then, we utilize $\overset{⌢}{H}$ to further extract the TF domain features. According to [38], by utilizing the symplectic finite Fourier transform (SFFT), we transform ${\overset{⌢}{H}}_{nt}$ into the DD domain, yielding the downlink CSI matrix ${\overset{⌢}{H}}_{\mathrm{DD},nt}\in C^{M\times N}$, i.e.,

(15) $$\begin{array}{cccc} & & {\overset{⌢}{H}}_{\mathrm{DD},nt}=F_{M}^{H}{\overset{⌢}{H}}_{n_{t}}F_{N}. & \end{array}$$

By sensing the instantaneous UE speed, the Doppler boundary $b_{\mathrm{Doppler}}$ can be obtained according to Equations (6) and (7). Consequently, we can perform denoising processing for ${\overset{⌢}{H}}_{\mathrm{DD},nt}$ along the Doppler axis with zeroing operations, thereby eliminating erroneous estimates outside the Doppler boundaries. Similarly, leveraging the reciprocity of the uplink and downlink path delays, zero-index CSI (the zero index represents the position in the support set, where the value is 0 [35,36]) can also be zeroed based on the delay support set $\left\{w_{\mathrm{dl},n_{t}}\right\}$, thus forming denoising processing for ${\overset{⌢}{H}}_{\mathrm{DD},nt}$ along the delay axis. Consequently, the enhanced downlink CSI in the DD domain, denoted as ${\hat{H}}_{\mathrm{DD},n_{t}}\in C^{M\times N}$, is expressed as

(16) $$\begin{array}{ccc} & & {\hat{H}}_{\mathrm{DD},n_{t}}=\left[\begin{array}{cc} 0_{M\times \left(a-1\right)} & {\overset{⌢}{H}}_{\mathrm{DD},nt} \end{array}\left(:,a:b\right)\;0_{M\times a}\right]⊙W_{\mathrm{dl},n_{t}}^{T}, \end{array}$$

where $a=\frac{N}{2}-b_{\mathrm{Doppler}}$, $b=\frac{N}{2}+b_{\mathrm{Doppler}}$, and $W_{\mathrm{dl},n_{t}}=1_{N\times 1}⊗w_{\mathrm{dl},n_{t}}$. By using inverse symplectic finite Fourier transform (ISFFT), the downlink CSI matrix in the TF domain, denoted as ${\hat{H}}_{n_{t}}\in C^{M\times N}$, is provided by

(17) $$\begin{array}{cccc} & & {\hat{H}}_{n_{t}}=F_{M}{\hat{H}}_{\mathrm{DD},n_{t}}F_{N}^{H}. & \end{array}$$

With the estimated ${\hat{H}}_{1},{\hat{H}}_{2},\cdots ,{\hat{H}}_{N_{t}}$, we form a three-dimensional CSI matrix $\overset{⌣}{H}\in C^{N_{t}\times M\times N}$ according to

(18) $$\begin{array}{cccc} & & \overset{⌣}{H}=\left[{\hat{H}}_{1},{\hat{H}}_{2},\cdots ,{\hat{H}}_{N_{t}}\right]. & \end{array}$$

For the *n*-th downlink CSI matrix with n=1,2,…,N, its spatial–frequency dimensions of $\overset{⌣}{H}$, denoted as ${\hat{H}}_{n}\in C^{N_{t}\times M}$, are extracted by ${\hat{H}}_{n}=\overset{⌣}{H}\left(:,:,n\right)$. For convenience and consistency with the CSI form of feedback, we omit the subscript *n* of ${\hat{H}}_{n}\in C^{N_{t}\times M}$, i.e.,

(19) $$\begin{array}{cccc} & & \hat{H}={\hat{H}}_{n}=\overset{⌣}{H}\left(:,:,n\right). & \end{array}$$

To eliminate the impact of downlink CSI on the detection of UL-DS, $\hat{H}$ is vectorized to $\hat{x}\in C^{1\times N_{t}M}$ according to $\hat{x}=\mathrm{vec}\left(\hat{H}\right)$. Then, with Y and $\hat{x}$, the SIS is used for preprocessing, i.e.,

(20) Y^=Y−ρEρENtMNtMgx^QT.

Subsequently, MMSE detection is used to obtain the detected UL-DS $\hat{d}\in C^{1\times N_{d}}$, which is expressed as

(21) $$\begin{array}{cccc} & & \hat{d}=\mathrm{dec}\left\{\frac{1}{\sqrt{\left(1-\rho \right)E}}{\left[g^{H}g+\frac{1}{\left(1-\rho \right)E}\sigma^{2}\right]}^{-1}g^{H}\hat{Y}\right\}, & \end{array}$$

where $\mathrm{dec}\left\{\cdot \right\}$ denotes the hard decision operation of UL-DS detection. It is worth noting that other detection methods, e.g., zero forcing (ZF) detection, could also be adopted.

In order to improve the recovery performance of downlink CSI and UL-DS, we utilize $I_{\mathrm{iter}}$ iterations, as employed in [8], while denoising processing is performed in the DD domain. Specifically, SIS is used to eliminate the impact of UL-DS on downlink CSI, i.e.,

(22) $$\begin{array}{cccc} & & \tilde{Y}=Y-\sqrt{\left(1-\rho \right)E}g\hat{d}. & \end{array}$$

By referencing [8], we employ iterative processing to suppress the superimposed interference. Then, the MMSE estimation of downlink CSI in Equation (13) is updated as

(23) $$\begin{array}{cccc} & & \tilde{x}=\sqrt{\frac{N_{t}M}{\rho E}}\frac{1}{N_{d}}{\left(g^{H}g+\frac{N_{t}M}{\rho EN_{d}}\sigma^{2}\right)}^{-1}g^{H}\tilde{Y}Q. & \end{array}$$

By iteratively performing Equations (22), (23), and (14)–(21), the downlink CSI and UL-DS are updated and optimized, leading to enhanced recovery performance. The detailed iteration algorithm is summarized in Algorithm 2. The proposed method significantly improves the accuracy of downlink CSI recovery and UL-DS detection while requiring only slightly additional computational complexity. This is validated according to computational complexity analysis and experiment results in Section 4 and Section 5.
**Algorithm 2** Downlink CSI and UL-DS recoveries.***Input*****:**  The received Y, Doppler boundary $b_{\mathrm{Doppler}}$, delay support set $\left\{w_{\mathrm{dl},n_{t}}\right\}$, and iteration number $I_{\mathrm{iter}}$.**for**$\;i=1,2,\ldots ,I_{\mathrm{iter}}$ **do**    **for** n=1,2,…,N **do**        **if** i=1 **then**           Utilize Equation (13) to estimate downlink CSI $\tilde{x}$.        **else**           Estimate the downlink CSI $\tilde{x}$ by using Equation (23).        **end if**    **end for**    Stack *N* downlink CSI matrices according to Equation (14).    **for** $n_{t}=1,2,\ldots ,N_{t}$ **do**        From Equations (15)–(17), perform denoising processing in the DD domain by exploiting Doppler boundary $b_{\mathrm{Doppler}}$ and delay support set $\left\{w_{\mathrm{dl},n_{t}}\right\}$.    **end for**    Use Equation (18) to achieve an enhanced downlink CSI $\overset{⌣}{H}$.    **for** n=1,2,…,N **do**        Obtain the recovered $\hat{H}$ by utilizing Equation (19).        Perform CSI SIS according to Equation (20).        Obtain the detected $\hat{d}$ by using Equation (21).        **if** $i<I_{\mathrm{iter}}$ **then**           Perform UL-DS SIS according to Equation (22).        **end if**    **end for****end for*****Output*****:**  The recovered $\hat{H}$ and $\hat{d}$.

**Remark** **2.** *There are other interference suppression methods that can also be used to recover downlink CSI and UL-DS, especially ML-based methods. In this paper, we take the MMSE method as an example to elaborate the recovery process of the proposed method, wherein an ML-based approach is not employed. The aim is to highlight the role of sensing information without integrating it into ML, making it difficult to distinguish whether it is the effect of sensing information or the effect produced by ML. Nonetheless, emerging ML-based CSI feedback methods (e.g., [12]) can also incorporate the methodology proposed in this paper. We validate this adaptation by using a simulation.*

## 4. Computational Complexity Analysis

According to [39], complex multiplication (CM) is used to measure the computational complexity, which also reflects the real-time feasibility and energy consumption of each comparison method. For expression convenience, “Xucla”, “ELM”, “LoSSen”, and “Prop” are utilized to denote the method in [8,12,40], and the proposed method, respectively. To further display the simple processing and slightly increased computational complexity of “Prop”, the denoising process of “Prop” is applied to the ML method in “ELM”, denoted as “Prop(ML)”. The comparison of computational complexity for the downlink CSI and UL-DS recoveries is provided in Table 1.

In the proposed method, SFFT, denoising processing, and ISFFT have the computational complexity of 2M2N+2MN2+MNMN22NtIiter. By jointly considering the one CSI coarse estimation, $I_{\mathrm{iter}}$ CSI SISs, $I_{\mathrm{iter}}$ UL-DS detections, $\left(I_{\mathrm{iter}}-1\right)$ UL-DS SISs, and $\left(I_{\mathrm{iter}}-1\right)$ CSI estimations, the computational complexity is $N+\left(N_{t}^{2}N_{d}M+N_{t}^{2}M+N_{t}N_{d}M+4N_{t}N_{d}+N_{t}M/2+2N_{t}+N_{d}/2+9/2\right)NI_{\mathrm{iter}}-3N_{t}N_{d}N/2$, which is equivalent to the CMs of “Xucla”. For the ML method in “ELM”, the computational complexity is (Nt2NdM+Nt2M+5NtNd5NtNd22+5Nt5Nt22+NdNd22+7/4)N+NdNtMN(12Iiter+1)+(2NdNtM+12Nd2+Nd+1/2)N(Iiter−1), without considering the training process. For the line-of-sight (LoS) sensing-based method in “LoSSen”, the computational complexity is $N+\left(N_{t}^{2}N_{d}M+N_{t}^{2}M+N_{t}N_{d}M+4N_{t}N_{d}+N_{t}M/2+2N_{t}+N_{d}/2+9/2\right)NI_{\mathrm{iter}}-3N_{t}N_{d}N/2+8N_{t}^{2}M^{2}$, with the training process similarly not taken into account. By conducting one SFFT, denoising processing, and ISSFT on the result of the ML method, the extra computational complexity is 2M2N+2MN2+MNMN22Nt. That is, with simple processing, the increased computational complexities of “Prop” and “Prop(ML)” are 2M2N+2MN2+MNMN22NtIiter and 2M2N+2MN2+MNMN22Nt, respectively.

For the case where $N_{t}=64$, M=8, N=8, $N_{d}=1024$, and $I_{\mathrm{iter}}=3$, the computational complexities in “Xucla”, “ELM”, “Prop(ML)”, and “Prop” are 824,202,356, 643,323,158, 643,456,278, and 824,601,716, respectively, whereas the computational complexities in “LoSSen” are 840,979,572. Compared with “Xucla” and “ELM”, the CMs of “Prop” and “Prop(ML)” increase by 0.0485% and 0.0207%, respectively.

Therefore, based on prior information, the proposed method conducting denoising processing only requires simple processing and slightly increased computational complexity. This indicates that the proposed method requires only a slight increase in energy consumption to significantly enhance the recovery accuracy of both downlink CSI and UL-DS, demonstrating its feasibility for real-time applications. The subsequent significant performance improvement is elaborated in Section 5.

## 5. Experiment Results

In this section, we present our numerical results to evaluate the performance of the proposed method. Definitions and basic parameters involved in the simulations are provided in Section 5.1. Subsequently, to verify the effectiveness of the proposed method, the normalized mean squared error (NMSE) of the recovered downlink CSI and bit error rate (BER) of the detected UL-DS are provided in Section 5.2. Finally, the robustness of the proposed method is presented in Section 5.3.

### 5.1. Parameters Setting

The definitions and basic parameters involved in simulations are presented as follows: $N_{t}=64$, M=8, N=8, $N_{d}=1024$, ρ=0.10, v=300 km/h, and $I_{\mathrm{iter}}=3$. The downlink carrier frequency is $f_{c}=4\;\mathrm{GHz}$, and the uplink carrier frequency is 3.7 GHz. The symbol duration and subcarrier spacing are set as T=1/15ms and Δf=15kHz, respectively. Thus, TΔf=1. The extended vehicular channel model-A (EVA) [41,42] is employed to generate CSI matrices for integer delay–Doppler cases, where a Doppler shift follows the classic Jakes spectrum [32]. The UE location coordinates (i.e., $\left(x_{t},\;y_{t}\right)$ and $\left(x_{t+\Delta t},\;y_{t+\Delta t},\right)$) provided by GNSS data are randomly generated based on the UE’s speed. The equivalent signal-to-noise ratio (SNR), NMSE, and BER are defined similarly to [12]. It is important to highlight that the proposed method is broadly applicable to CSI-dependent scenarios, including vehicle-to-everything (V2X) and wireless fidelity (WiFi) systems, with no specific limitations.

“Prop” performs the denoising in both the delay and Doppler dimensions. To verify the effectiveness of prior information, we conduct simulations for the proposed method, wherein denoising is exclusively performed along either the delay or Doppler dimension. Consequently, we use “Prop (without delay)” and “Prop (without Doppler)” to denote the cases where denoising occurs solely in the Doppler and delay dimensions, respectively.

To further verify the effectiveness of “Prop” when applied in ML-based CSI feedback, we apply the denoising process of “Prop” to the simulation of the ML method outlined in “ELM”, denoted as “Prop(ML)”. This is beneficial for proving that the proposed method of utilizing inactive sensing information can also be applied to emerging ML-based CSI feedback methods.

### 5.2. Effectiveness Analysis

**Figure 2:**
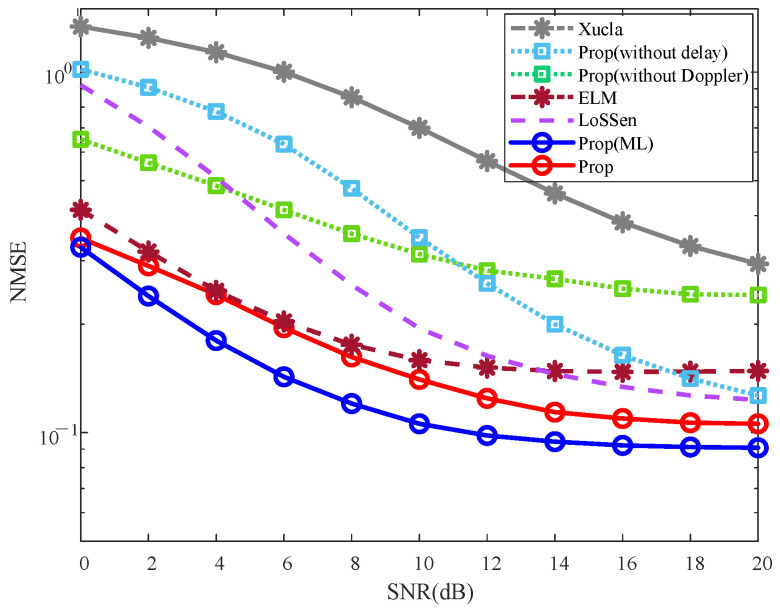
NMSE effectiveness, where ρ=0.10, v=300 km/h, and $I_{\mathrm{iter}}=3$.

**Figure 3:**
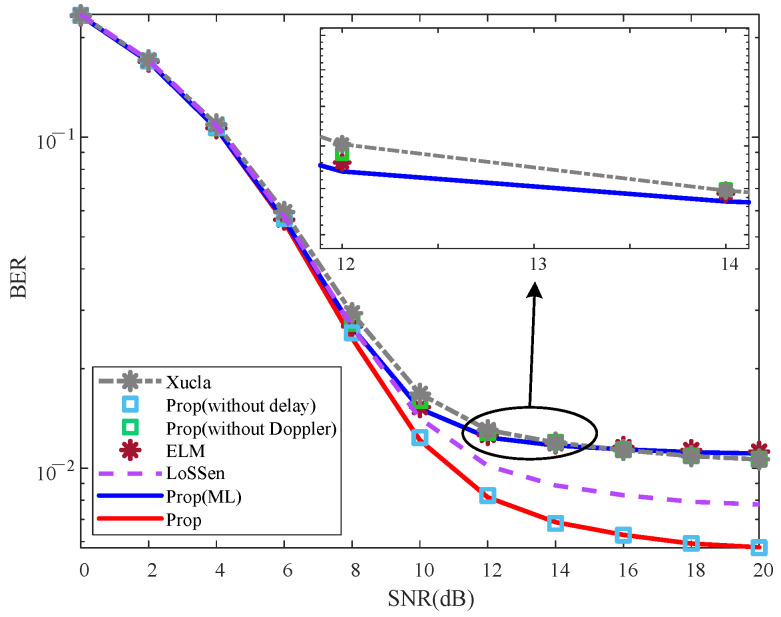
BER effectiveness, where ρ=0.10, v=300 km/h, and $I_{\mathrm{iter}}=3$.

To validate the effectiveness of the proposed method, the NMSE and BER curves are plotted in Figure 2 and Figure 3, respectively.

#### 5.2.1. NMSE Effectiveness Analysis

As depicted in Figure 2, the NMSE of “Prop” is consistently smaller than those of “Xucla”, “ELM”, “LoSSen”, “Prop (without delay)”, and “Prop (without Doppler)” for each given SNR. This improvement is attributed to the denoising process that leverages prior information in the DD domain. Specifically, the NMSE of “Prop” is smaller than those of “Prop (without delay)” and “Prop (without Doppler)”, indicating the effectiveness of multiple prior information fusion. The NMSEs of “Prop (without delay)” and “Prop (without Doppler)” are both smaller than that of “Xucla”, which confirms that both the Doppler shift prior from the inactive sensing information and the path delay prior from the channel reciprocity are effective in suppressing interference. In addition, “Prop(ML)” outperforms “ELM” in terms of NMSE, further verifying the effectiveness of the proposed method in the application of ML-based CSI feedback. Overall, the proposed method proves to be advantageous in improving the NMSE performance in various SNR scenarios.

#### 5.2.2. BER Effectiveness Analysis

From Figure 3, it is evident that the BERs of “Xucla”, “ELM”, and “LoSSen” are higher than that of “Prop” for each specific SNR. The reason is that “Prop” improves the recovery performance of downlink CSI through the additional prior information, thereby enhancing the detection performance of UL-DS due to the superimposed mode and SIS processing. Furthermore, the BERs of “Prop (without Doppler)” and “Prop (without delay)” are similar to those of “Xucla” and “Prop”, respectively. This indicates that the Doppler shift prior information introduced by the inactive sensing information from the new modal data is significantly effective for the UL-DS detection in the classical superimposed CSI feedback method. Additionally, “Prop(ML)” outperforms “ELM” slightly in terms of BER, further affirming the effectiveness of the proposed method. On the whole, compared with “Xucla”, “ELM”, and “LoSSen”, the proposed method effectively reduces the BER of UL-DS.

### 5.3. Robustness Analysis

In this section, the power proportional coefficient ρ and the UE’s speed *v* are employed to analyze the robustness, as they directly affect the superimposed interference and the accuracy of sensing assistance. To analyze the robustness of “Prop” against parameter variations, only the analyzed parameter (either ρ or *v*) is varied, while keeping all other basic parameters the same as those specified in Section 5.1.

#### 5.3.1. Robustness Against ρ

**Figure 4:**
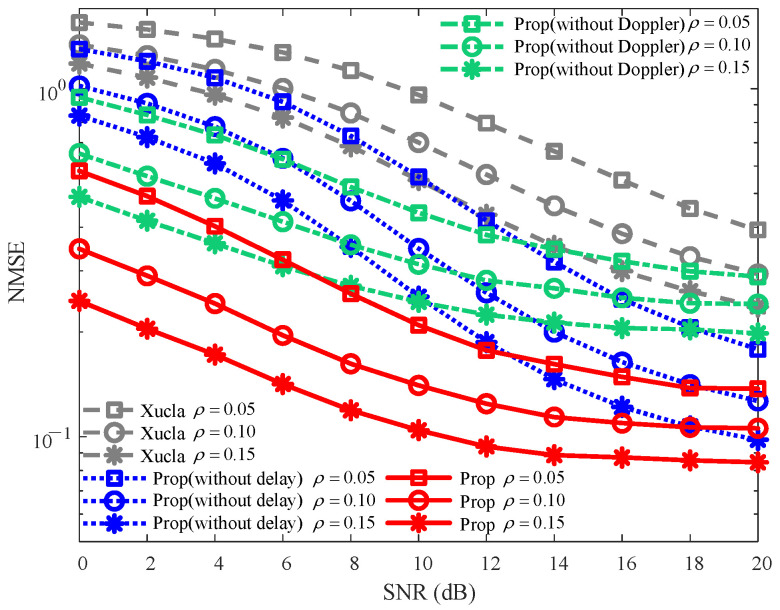
NMSE robustness against varying ρ, where v=300 km/h.

The NMSE curves against varying ρ (i.e., ρ=0.05, ρ=0.10, and ρ=0.15) are plotted in Figure 4. For each given ρ, the NMSEs of “Prop” are consistently smaller than those of “Xucla”, “Prop (without delay)”, and “Prop (without Doppler)” across the entire SNR range. With the increase in ρ, the NMSEs decrease for “Xucla”, “Prop (without delay)”, “Prop (without Doppler)”, and “Prop”, and vice versa. This behavior arises because ρ, as the power proportional coefficient of the downlink CSI, dictates that a larger value results in the allocation of more transmission power to downlink CSI, leading to improved CSI recovery at the BS. Overall, against the variations in ρ, “Prop” continues to outperform “Xucla”, “Prop (without delay)”, and “Prop (without Doppler)” in terms of NMSE.

**Figure 5:**
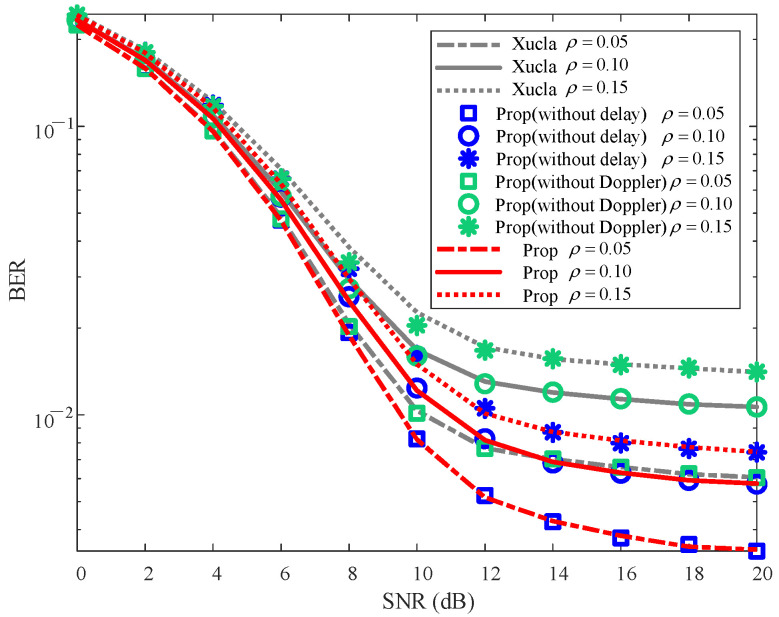
BER robustness against varying ρ, where v=300 km/h.

Figure 5 depicts the BER performance for the UL-DS against the variations in ρ. It can be observed that the BERs of “Prop” and “Prop (without delay)” are similar and smaller than those of “Xucla” and “Prop (without Doppler)” for each given ρ and SNR. With the increase in ρ, the BERs of “Xucla”, “Prop (without delay)”, “Prop (without Doppler)”, and “Prop” increase, and vice versa. The reason for this behavior is that a higher value of ρ results in an increased allocation of power to the downlink CSI (at the expense of UL-DS), which deteriorates the detection performance of UL-DS. Nevertheless, “Prop” and “Prop (without delay)” still obtain smaller BERs than those of “Xucla” and “Prop (without Doppler)”. Thus, against the impact of ρ, the proposed method improves the BER performance compared with “Xucla” and “Prop (without Doppler)”.

#### 5.3.2. Robustness Against *v*

**Figure 6:**
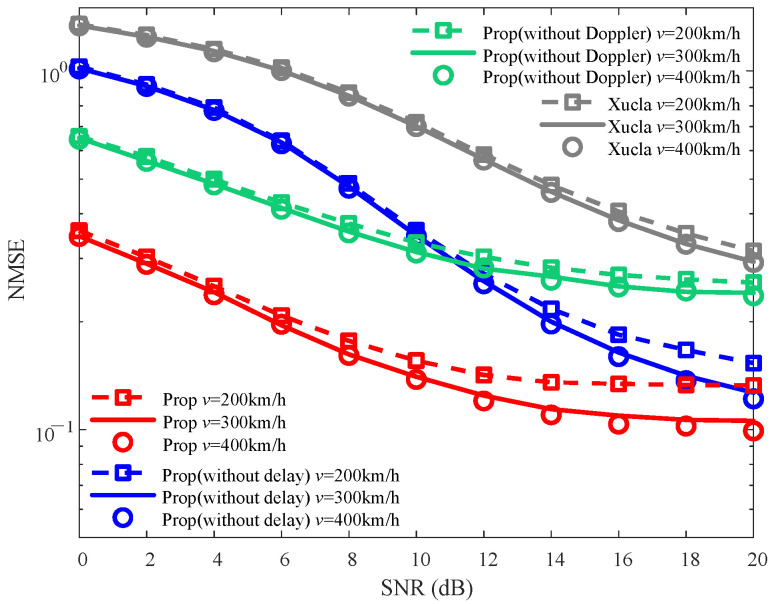
NMSE robustness against varying *v*, where ρ=0.10.

The impact of varying *v* (i.e., v=200 km/h, v=300 km/h, and v=400 km/h) on the NMSE curves is presented in Figure 6. From Figure 6, it is evident that for each given *v* across the entire SNR range, the NMSEs of “Prop” are smaller than those of “Xucla”, “Prop (without delay)”, and “Prop (without Doppler)”. With the decrease in *v*, the NMSEs of “Xucla” and “Prop (without Doppler)” are changed slightly. The reason is that the speed *v* affects the Doppler shift, while the Doppler shift prior information is not utilized in “Xucla” and “Prop (without Doppler)”. On the contrary, for “Prop” and “Prop (without delay)”, the NMSEs decrease with the increase in *v*, and vice versa. This behavior is attributed to the fact that increased *v* leads to a larger Doppler shift in the downlink CSI, which is closer to the pre-defined Doppler boundary value at the given resolution of the path Doppler shifts. Thus, the denoising processing utilizing the Doppler shift prior information achieves a higher accuracy for interference suppression in the Doppler dimension. On the whole, against the variations in *v*, the NMSEs of “Xucla”, “Prop (without delay)”, and “Prop (without Doppler)” are reduced by using “Prop”.

**Figure 7:**
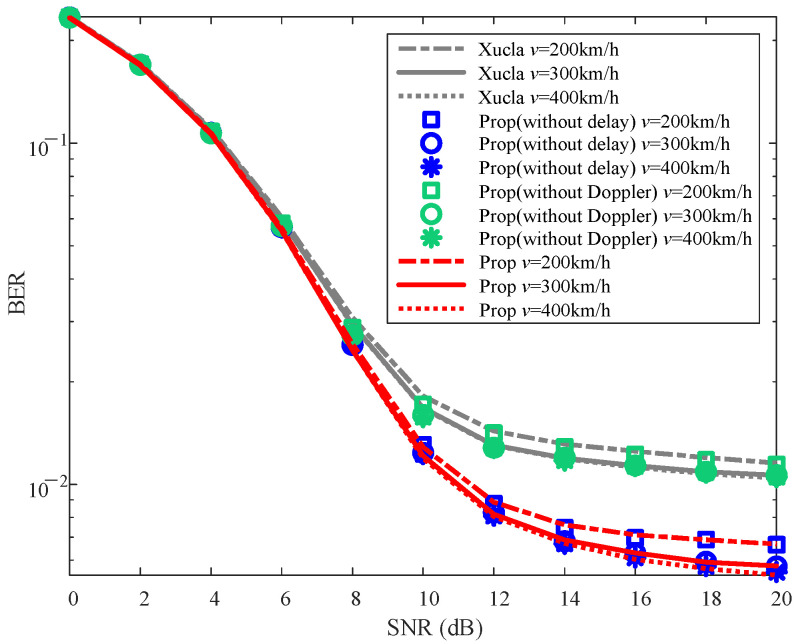
BER robustness against varying *v*, where ρ=0.10.

To verify the BER performance against the impact of *v*, Figure 7 plots the BER curves. For each given *v* and SNR, the BER values of “Prop” and “Prop (without delay)” are similar and smaller than those of “Xucla” and “Prop (without Doppler)”. As observed from Figure 7, with the increase in *v*, the changes in BER of “Xucla” and “Prop (without Doppler)” are not readily apparent. On the contrary, the BERs of “Prop” and “Prop (without delay)” decrease as *v* increases, and this could be interpreted as the proposed method improving the BER performance via refining the recovery accuracy of the downlink CSI. Namely, “Prop” and “Prop (without delay)” refine the recovery accuracy of the downlink CSI by denoising processing that utilizes the Doppler shift prior information, and then use the refined downlink CSI for SIS to recover UL-DS. Therefore, an increase in *v* has the same impact on the BER of UL-DS as it does on the NMSE of downlink CSI. In a word, when compared with “Xucla” and “Prop (without Doppler)”, the proposed method improves the BER performance against the impact of *v*.

## 6. Conclusions

The proposed superimposed CSI feedback method assisted by inactive sensing information improves the recovery accuracy without extra hardware overhead. On the one hand, the CSI feedback overhead and the spectral efficiency are improved by adopting the superimposed mode to avoid uplink transmission resource occupation. On the other hand, inspired by sensing-assisted communication, we extract the location, speed, and path information by utilizing the inactive sensing information at the BS, which alleviates the burden on system resources. Different from communication signals for CSI feedback, a new modal data is utilized to derive additional prior information for interference suppression. Leveraging the derived prior information, we conduct the denoising process in both the delay and Doppler dimensions of downlink CSI in the DD domain. The proposed method demonstrates superior performance in the downlink CSI and UL-DS recoveries, whether based on the classical method or the ML-based method. Simulation results affirm the effectiveness of the proposed feedback method and its robustness against parameter variations. However, for the sake of simplicity, this work assumes on-grid channel parameters. In practice, due to limited transmission resources, as well as the limited computing resources and battery energy of the UE, the off-grid effect remains a significant challenge. Furthermore, the scenarios where sensing data contains errors or delays are not considered. In our future work, we will investigate sensing-assisted CSI feedback for off-grid conditions and develop methods that incorporate GNSS error models to enhance CSI reconstruction accuracy, thereby making it more suitable for practical applications.

## Figures and Tables

**Table 1:** The analysis of computational complexity.

Method	Computational Complexity	Computational Complexity Case	Increased Complexity
Xucla	N+Nt2NdM+Nt2M+NtNdM+4NtNd+NtMNtM22+2Nt+NdNd22+9/2NIiter −3NtNdN3NtNdN22	824,202,356	−
ELM	Nt2NdM+Nt2M+5NtNd5NtNd22+5Nt5Nt22+NdNd22+7/4N+NdNtMN12Iiter+1 $+\left(2N_{d}N_{t}M+12N_{d}^{2}+N_{d}+1/2\;\right)N\left(I_{\mathrm{iter}}-1\right)$	643,323,158	−
LoSSen	N+Nt2NdM+Nt2M+NtNdM+4NtNd+NtMNtM22+2Nt+NdNd22+9/2NIiter $-3N_{t}N_{d}N/2+8N_{t}^{2}M^{2}N$	840,979,572	−
Prop(ML)	Nt2NdM+Nt2M+5NtNd5NtNd22+5Nt5Nt22+NdNd22+7/4N+NdNtMN12Iiter+1 +2NdNtM+12Nd2+Nd+1/2NIiter−1+2M2N+2MN2+MNMN22Nt	643,456,278	0.0207%
Prop	N+Nt2NdM+Nt2M+NtNdM+4NtNd+NtMNtM22+2Nt+NdNd22+9/2NIiter −3NtNdN3NtNdN22+2M2N+2MN2+MNMN22NtIiter	824,601,716	0.0485%

## Data Availability

All data generated or analyzed during this study are included in the published article.
